# Material Anisotropy in Additively Manufactured Polymers and Polymer Composites: A Review

**DOI:** 10.3390/polym13193368

**Published:** 2021-09-30

**Authors:** Nima Zohdi, Richard (Chunhui) Yang

**Affiliations:** School of Engineering, Design and Built Environment, Western Sydney University, Penrith, NSW 2751, Australia; n.zohdi@westernsydney.edu.au

**Keywords:** additive manufacturing, fused deposition modelling (FDM), material anisotropy, mechanical anisotropy, thermal anisotropy, electrical anisotropy

## Abstract

Additive manufacturing (AM) is a sustainable and innovative manufacturing technology to fabricate products with specific properties and complex shapes for additive manufacturable materials including polymers, steels, titanium, copper, ceramics, composites, etc. This technology can well facilitate consumer needs on products with complex geometry and shape, high strength and lightweight. It is sustainable with having a layer-by-layer manufacturing process contrary to the traditional material removal technology—subtractive manufacturing. However, there are still challenges on the AM technologies, which created barriers for their further applications in engineering fields. For example, materials properties including mechanical, electrical, and thermal properties of the additively manufactured products are greatly affected by using different ways of AM methods and it was found as the material anisotropy phenomenon. In this study, a detailed literature review is conducted to investigate research work conducted on the material anisotropy phenomenon of additively manufactured materials. Based on research findings on material anisotropy phenomenon reported in the literature, this review paper aims to understand the nature of this phenomenon, address main factors and parameters influencing its severity on thermal, electrical and mechanical properties of 3D printed parts, and also, explore potential methods to minimise or mitigate this unwanted anisotropy. The outcomes of this study would be able to shed a light on improving additive manufacturing technologies and material properties of additively manufactured materials.

## 1. Introduction

Additive manufacturing (AM) is a process of printing successive layers of materials on top of each other to form a final product [1]. This process was firstly introduced in 1986 by Hull [2]. A major benefit of adopting AM to conventional manufacturing technologies, including moulding injection, extrusion and subtractive manufacturing is its capability to produce products with much more complex shapes and geometries meanwhile it can significantly reduce the amount of waste [3,4]. In this manufacturing technology, a meshed 3D computer model can be developed by computer-aided design (CAD) software in a format of Surface Tessellation Language (STL) file and then it will be input into the 3D printer. With its embedded 3D printing software, the model with mesh data will be sliced into a build file of 2D layers before feeding to the 3D printing machine to do the printing [5]. Different methods of AM were developed as namely fused deposition modelling (FDM), powder bed and inkjet head 3D printing (3DP), stereolithography (SLA), three-dimensional printing (3DP), digital light processing (DLP), selective laser sintering (SLS), laminated object manufacturing (LOM), polyjet and paste extrusion printing or direct writing 3D printing (DW), etc. For the past decade, this process found its way into many industries and fields such as producing lightweight parts, components, structures and systems for the automotive and aerospace industry, architectural modelling for presenting the functionality of the parts, medical application for replacing fractured bones with implants and improving the manufacturing of fuel cells by producing very thin layers of materials platinum [6,7].

Many investigations showed the capabilities of additive manufacturing parts with different materials ranging from polymers and polymer nanocomposites to metals, ceramics and even concretes [1,7]. Amongst the materials mentioned above, polymers are the most popular material utilised to produce parts by using AM. Despite the popularity of AM in producing geometrically complex parts, challenges such as relatively lower mechanical strength and lower thermal and electrical conductivity properties were reported when samples were being printed in different build orientations. Moreover, weak inter-layer adhesion, as well as air voids produced between rasters and layers, are the two most challenging issues for additively manufactured polymeric parts by using DLP, SLS and FDM. These gaps are known as the spaces between rasters or roads in FDM printing during the fabrication of polymer parts and can potentially decrease mechanical properties of the parts such as tensile ultimate and yield strengths compared to mould injected and thermal pressed parts. As for additively manufactured polymer parts under loadings, there is a fibre delamination-like failure mode, which can happen along the raster or layer direction due to weak inter-layer adhesion. This can make the printed parts weak in loadings perpendicular to the build orientation, which is known as transverse orientation. This phenomenon can be further reflected in the material properties of the parts to show anisotropy when having different build orientations. It does not only affect the mechanical properties of these parts but also the existing internal air voids can act as thermal and electrical barriers and lower their thermal and electrical conductivities significantly.

**Figure 1:**
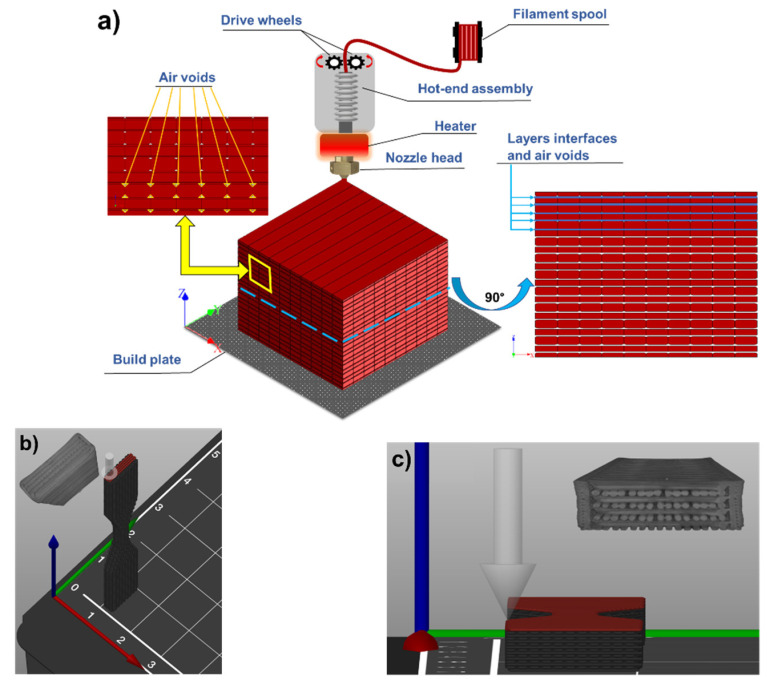
Schematic of FDM process: (**a**) FDM process and air voids formed throughout the sample; (**b**) transverse build orientation; and (**c**) longitudinal build orientation.

Figure 1a shows the schematic of a typical FDM process, which depicts the main components of a 3D printing machine as well as the air voids that form throughout the printed sample at their layer interfaces. Figure 1b,c also show the two major build orientations of transverse and longitudinal and their associated cross-sectional CT scan images, respectively.

To the best knowledge of authors, only one review paper was published so far on the anisotropy of 3D printed metal parts [8] but no comprehensive review paper on this phenomenon for polymer parts is available. A review paper published by Gao et al. [9] provides an understanding of the process-structure properties of interlayer bonds and their effects on the mechanical anisotropy in the FDM technique. While the paper clarifies the state of the knowledge on mechanical anisotropy and fundamentals of the phenomenon, no information on the anisotropy of other material properties were provided in it. A similar focus was adopted by Goh et al. but anisotropy of the parts was briefly discussed [10]. The characterisation of the mechanical properties of printed parts and the relative theoretical and computational approaches were also reviewed by Cuan-Uruizo et al. [11], but little focus on anisotropy motivated the authors to create this review paper.

The current paper reviews published literatures that investigated the fundamentals of anisotropy and its effects on mechanical, electrical and thermal properties of 3D printed parts. This review also provides relevant information on the methods developed so far to minimise or mitigate this effect in FDM manufactured parts. The technical barriers and challenges are identified for future work. This review paper is structured with the following sections: Section 2 provides the fundamental information on anisotropy amongst additive manufacturing methods specifically targeting the methods with the highest possibilities of developing the symptoms. This section will also cover the information on the anisotropy behaviour on mechanical strength as well as thermal and electrical conductivities. In Section 3, influential factors and parameters contributing to the material anisotropy and the methods that were developed so far to mitigate anisotropy are discussed. Section 4 addresses the research challenges and recommendations on the future works and in the last section, Section 5 draws conclusions to summarise the whole paper.

## 2. Material Anisotropy in Additively Manufactured Polymer Parts

Material Anisotropy can be found in many polymeric parts produced via different technologies of additive manufacturing such as FDM, SLS, DLP and SLA [12,13,14,15,16] and the material anisotropy is defined in this study to have three categories: (a) mechanical anisotropy; (b) electrical anisotropy and (c) thermal anisotropy.

The mechanical anisotropy reported for parts printed via the FDM method is the highest amongst all other AM methods and can go as high as almost 50%. This figure for SLS is around 10%, DLP around 5% and SLA is only around 1 per cent [14,17].

Although the materials manufactured by FDM showed the most anisotropic levels, few researchers reported some noticeable levels of mechanical anisotropy in parts printed by SLS [13,18,19,20]. For instance, Ajoku et al. reported the use of polyamide 12 (PA12) in producing parts with selected laser sintering (SLS). Their samples showed a discrepancy of 16 per cent in tensile strength and 11.2 per cent in modulus for parts printed in the X, Y, and Z axes leading to unequal and lower mechanical properties in parts printed in Z build orientation than X orientation [18]. Beitz et al. also reported a 48.6 per cent difference when build orientation was altered [19]. On the other hand, some other researchers reported no significant changes in the tensile strength of the parts printed by SLS [21]. Parameters such as laser power (density), layer thickness, laser beam speed, build and powder deposition orientations as well as layer thickness, refresh rate, bed temperature and hatch pattern were reported to be amongst the influential processing parameters on the properties of the printed parts by SLS method [13,14,17,18,19,22].

Other than the SLS method, material anisotropy was also reported in the production of polymeric parts by Digital Light Processing (DLP) method [14,23]. Monzón et al. indicated that samples without post-curing in both resins showed 5 per cent anisotropic behaviours in their mechanical properties when printed in transverse and longitudinal directions [14].

Anisotropy of material properties of the printed parts via FDM is so far the most significant issue [16,24]. Printed parts suffer from low mechanical, electrical and thermal properties towards the built or axial orientation (*Z*-axis) [25,26]. As it was mentioned earlier, mechanical anisotropy in parts printed by FDM could go as high as 50% depending on the layer size such as thickness, width, or diameter. Reports showed that FDM produced parts have also weaker thermal and electrical conductivity properties towards build orientation than other orientations [27,28,29,30].

Table 1 summarises the main factors and parameters of 3D printing material and process, which contribute to material anisotropy investigated by the researchers so far. The investigation of these factors and parameters helps improve mechanical, thermal, and electrical properties of the 3D printed parts and reduce the unwanted material anisotropy. The degree of importance was marked by stars per item, in which zero stars relate to the least important factor or parameter and 3 stars relates to the most important ones.

One significant factor causing the material anisotropy, in particular for mechanical anisotropy, is the insufficient interlayer bonding adhesion between adjacent rasters, which is a consequence of incomplete diffusion and neck growth between layers [40]. During the FDM printing, the thermoplastic filament is fed by drive wheels into the heating chamber and then is extruded through the nozzle upon its melting temperature; then solidified and deposited on the platform layer by layer. Although the deposition lines can be integrated into adjacent lines, inter and intralayer deformation occurs in the form of cracks between the cylindrical lines. The extruded material cools quickly from melting to printer chamber temperature, resulting in the development of inner stresses responsible for the weak bond between two deposition lines, which causes the delamination or part fabrication failure [1,17,30]. This structure inhomogeneity results in impaired mechanical strengths in the part produced via FDM.

**Figure 2:**
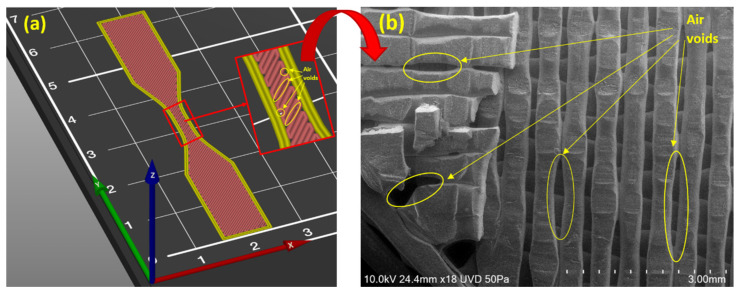
Void formation on samples printed by FDM: (**a**) 3D model showing the air voids; and (**b**) SEM image showing the air voids.

The other factor causing the anisotropy can be related to the significant air voids which form between adjacent cylindrical lines while printing [48]. Although these air voids can be reduced in size by changing the printing parameters like air gaps, the negative effect and the presence of these voids cannot be removed fully and that is the main challenge in parts produced via FDM [48]. Figure 2a represents a 3D model to show how the air voids form in each layer and Figure 2b is the SEM image of a fractured surface of a 3D printed part.

In addition, this kind of material anisotropy can also cause difficulties in product design and topology optimisation (TO) of complex designs such as load-bearing structures [49] however, the focus of this review is only on discussing the material properties, which were affected by anisotropy in 3D printed parts. Table 2 shows a summary of researches highlighting thermal, electrical, and mechanical anisotropies in parts printed via different AM methods.

### 2.1. Mechanical Anisotropy

In this study, the mechanical anisotropy represents the anisotropy phenomena of mechanical properties of the 3D printed materials, which were investigated extensively for mechanical properties including tensile strength, flexural strength, elongation, compression strength, shear strength, hardness, wear and friction [20,31,59,60,61,62]. Few researchers initially studied the existence of this anisotropy and its effects on the mechanical properties of the 3D printed parts [63,64]. Some of the contributing factors in mechanical anisotropy of parts printed by using the FDM method are known as air gaps/voids, build orientation and raster angle. However, alternations in build orientation are known to be the most influential factor to highlight the mechanical anisotropy amongst all. Other AM methods such as PolyJet [57] and stereolithography [58] also showed the mechanical anisotropy when changing the build orientations to some extend but FDM still was the most vulnerable method to build orientation changes.

Layer delamination and the failure of a part start from the free surface of the 3D printed part. The orientation and severity of the crack growth depend on the raster angle and intensity of the pores, respectively [61,65]. One way to limit the crack extension and damage to the structure of the parts printed via the FDM can change the raster angle from time to time. Misalignment of filaments can change pore alignments, therefore, cracks that tend to grow along the boundaries can be stopped from further propagation by these misalignments [61,66]. Although this alteration does influence the measurements parallel to the print deposition, the issue with the layer delamination remains when the measurement is perpendicular to the build orientation.

Baker et al. calculated the compressive modulus for FDM-built prototypes of polylactic acid (PLA) and acrylonitrile butadiene styrene (ABS) in two longitudinal and transverse orientations. The values were compared with the equivalent measured value for the bulk of materials [12]. According to their results, the compressive modulus for PLA in the Z direction (transverse) was around 27% less than the value measured for bulk material and the longitudinal orientation measured value was around 16.6% less than the value measured for bulk PLA. Moreover, the compressive elastic modulus for transverse build orientation was calculated around 9% lower than that for the longitudinal build orientation. Although the investigation using the compression test could represent the existence of anisotropy, more investigations on tensile strength as well as surface deformation behaviours are still needed.

A severe compression test condition was adopted by Guessasma et al. to embolden the mechanical anisotropy in FDM-printed parts [61]. It was found that mechanical anisotropy happened due to lateral damage extension in raster angles of 30°, 45° and 60° when the load was perpendicular to the build orientation.

Adding additives can potentially make improvements in reducing mechanical anisotropy. Adding additives such as TiO_2_, Jute and TPE elastomers to ABS polymer showed changes in mechanical anisotropy when parts were printed in two build orientations of longitudinal and transverse [67]. TPE was proven to help minimise the UTS gaps between the samples printed in two different build orientations. However, in this study, UTS dropped noticeably by almost 15.5 per cent when compared to the pure ABS samples. The results of the elongation at break showed a reduction in anisotropy for samples with TiO_2_ and TPE but the drawback was a huge drop in elongation at break when these results were compared with those of the pure ABS. In an interesting section of evaluating the cross-section SEM images, samples containing TiO_2_ showed much better bonds between rasters and represented lower air voids compared to those in the pure ABS. This finding also justified the 12.9 per cent improvement in UTS results for samples printed in longitudinal build orientation and 30.5 per cent in the samples printed in a transverse orientation.

In a recent study conducted by Prasong et al. [68], the influence of adding additives on the anisotropy of PLA parts printed via the FDM method was investigated. This study showed that by adding some particular additives such as poly(butylene succinate) (PBS) by 27 wt% to the mixture of PLA-nano talc and removing poly(butylene adipate-co-terephthalate) (PBAT) from the system, not only the tensile strength can be improved by 25% for longitudinally printed samples and 33% for transversely printed samples, but also the anisotropy in parts can be decreased by almost 5%. Although adding a small percentage of PBAT (around 9 wt%) caused a slight increase in toughness and a decrease in tensile strength values for samples printed in longitudinal and transverse build orientations but the anisotropy degree remained exactly the same.

In another study, Ding et al. [37] investigated the effect of adding carbon fibre on minimising the anisotropy in FDM-printed parts. Although layer thickness and print temperature were two effective factors in decreasing the anisotropy for parts printed in sideway and transverse build print orientations, the samples printed in longitudinal build orientation still showed a substantial difference when compared to those with the other build orientations.

Anisotropy level can also be changed when the type of the polymer is changed. S shear strength in ABS-M30 parts was studied by Balderrama-Armendariz et al. and they found a very negligible difference in ultimate shear strength and shear modulus when parts printed in longitudinal were compared to those printed in transverse orientation [62]. Moreover, the values of the ultimate strength study were showing much higher values than that of conventional ABS. This can conclude that the type of polymer can have a positive influence on the mechanical properties of a printed part.

**Figure 3:**
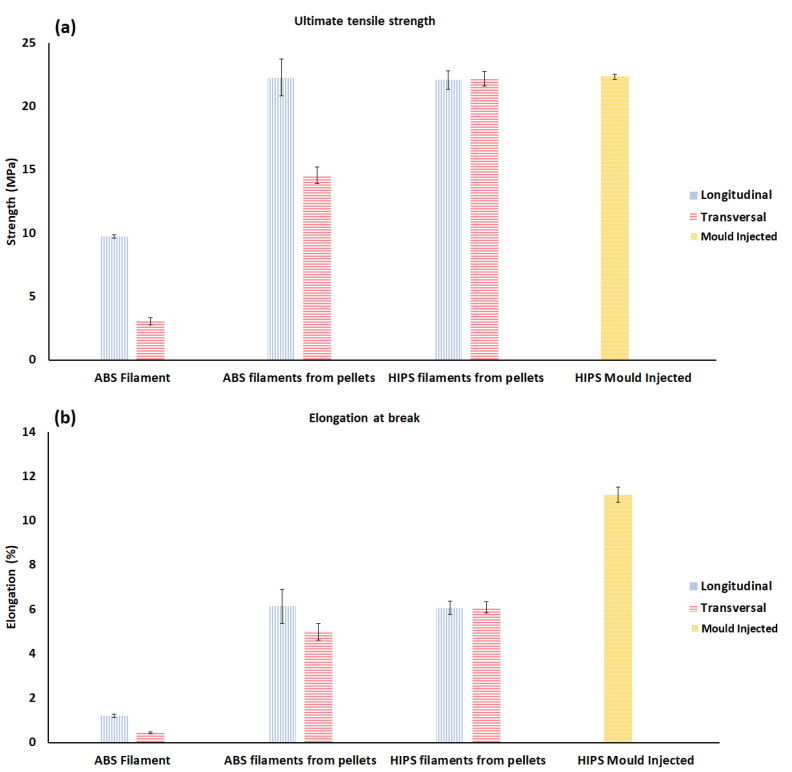
Tensile test results: (**a**) Ultimate tensile strength, and (**b**) Elongation at the break, in which the error bars represent the standard deviation of the samples. Reprinted with permission from Ref. [16]. 2019, GCSTMR.

In our previous study [16], the authors also investigated the mechanical anisotropy in parts printed with high impact polystyrene (HIPS) and acrylonitrile butadiene styrene (ABS). It was found that the samples printed in two different build orientations of longitudinal and transverse with HIPS showed minimal anisotropy when compared to ABS samples. SEM images proved lower layer adhesion in parts made of ABS polymers compared to HIPS polymers. In addition, the tensile strength of 3D printed HIPS was found much close to values for the mould injected samples which shows that by choosing a proper polymer, almost the same mechanical properties as those of the mould injected sample could be achieved. However, more studies on different printing temperatures, infill percentages and layer thickness are still required to find more optimum 3D printing processes. Figure 3 shows the influences of material selection on mitigating the mechanical anisotropy for parts printed via the FDM method.

### 2.2. Electrical Anisotropy

**Figure 4:**
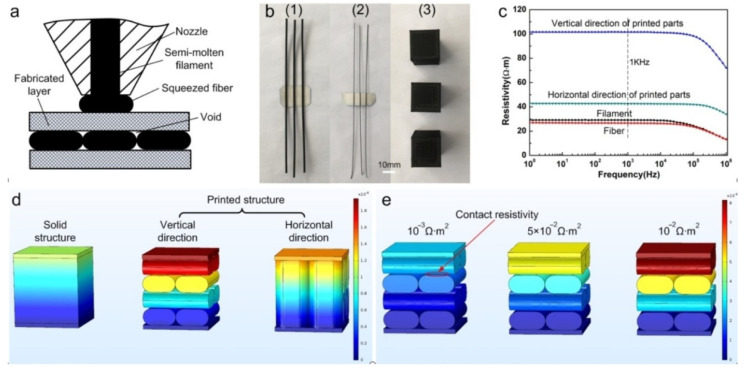
Different shapes and their resistivities for materials printed with the FDM method: (**a**) FDM printing process; (**b**) different materials shapes: filament, fibre, and cube; (**c**) resistivities of materials printed in different build orientations and frequencies of 1 Hz–1 MHz; (**d**) different structures of samples: solid, vertical direction and horizontal direction and (**e**) resistivities of the printed structure models in the vertical direction. Adapted with permission from Ref. [38]. 2017, MDPI.

The electrical anisotropy denotes the anisotropy in the electrical conductivity of 3D printed polymer composites, which was reported in several research works. Gnanasekaran et al. investigated the anisotropy of electrical properties for samples printed with different raster angles [28]. Based on their results, the measured electrical anisotropy printed with raster angles ranging from 0 to 90 degrees can vary enormously. For instance, electrical resistance for polybutylene terephthalate/carbon nanotube (PBT/CNT) samples, with a CNT volume fraction of 0.036, can increase up to 32 per cent when electrical resistance was measured perpendicular to the filament deposition. This difference could be related to the formation of interfaces between the adjacent line which can act as a resistance when samples were measured perpendicular to the printed rasters [28]. Similarly, in another study, samples printed in a vertical build orientation had resistivity equal to 3 to 4 times lower than the samples printed in a horizontal build orientation [38]. Figure 4 below shows the resistivity differences when the build orientation is changed.

**Figure 5:**
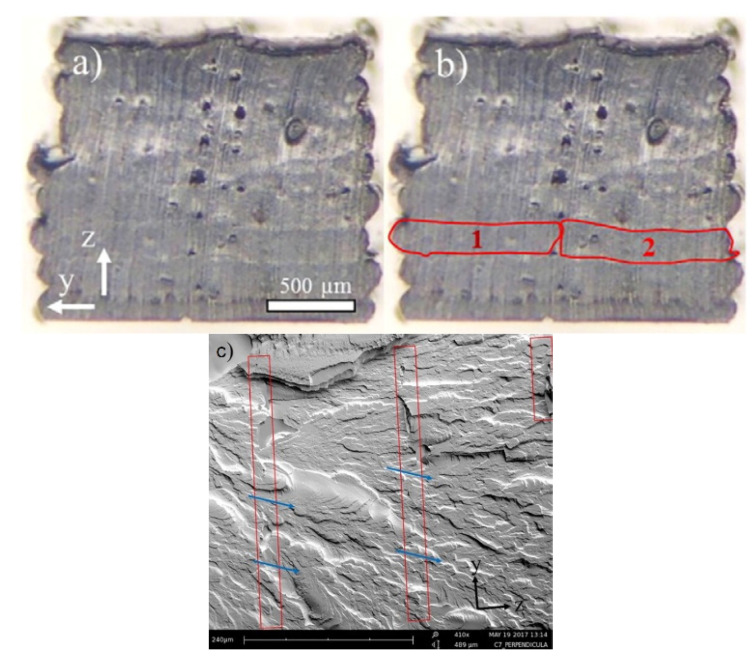
SEM images of (**a**) cross-section, (**b**) cross-section with highlighted layers in the *y*-direction and (**c**) showing the y-z view of the interlayer bonds. Adapted with permission from Ref. [69]. 2017, Elsevier.

In contrast, in another research work, adding multi-walled carbon nanotube to thermoplastic polyurethane (TPU/MWCNT) resulted in preserving the electrical conductivity measured in both through-layer and cross-layer directions [69]. This behaviour can be attributed to the consistent distribution of MWCNT and excellent interlayer adhesion to those reported in the literature. From the SEM images of the cross-section of 3D printed TPU/MWCNT composite as shown in Figure 5, the layer interface (highlighted by red lines) is almost not recognisable due to the superior adhesion between the layers.

Other than the build orientation, some machine parameters of the 3D printer such as raster width and air gap can be influential on forming electrical anisotropies. The most important reasons why resistivity differences happen in FDM-printed parts using are the air voids and the imperfect bonding conditions that form between adjacent rasters [38,70]. On top of these parameters, the formation of the conductive fillers along the raster deposition can also greatly influence the values of the electrical conductivity [71].

In the stereolithography printing method, mechanical anisotropy is known to be negligible as the strong bonds can happen due to the polymerisation reactions [72]. However, this is not the case for electrical anisotropy. Chung et al. investigated the electrical anisotropy in stereolithography-printed parts [73]. They traced changes in electrical permittivity when the printing direction was altered. They also investigated other variables such as layer thickness and layer printing sequence on the molecular structure of the printed parts. They found that the in-plane capacitance is dependent on the printed layer sequence and can vary when comparing the capacitance of the first layer and the last layer.

Thaler et al. also investigated the electrical anisotropy in their FDM-printed samples [71]. ABS-CNT samples were made with different percentages of the filler and in-layer and the through-layer electrical conductivity of the printed samples were compared with those of the compression moulded samples. The obtained results suggested the electrical conductivity of the compression moulded samples was found as the highest for the same percentages of the additives followed by in-layer and through-layer. Unlike previous studies that air voids and poor bonding between layers were counted to be amongst the main reasons for the conductivity changes, they tried to consider a different approach. They claimed that the differences between compression moulded and in-layer results happened because of the in-layer CNT orientation that happened due to the applied shear forces while printing. Moreover, they claimed that the CNT orientation prevents the percolative network to form and which could be the reason for conductivity drop in in-layer measurements compared to that in the through-layer conductivity measurement. As the anisotropy for in-layer and through-layer measurements in higher CNT concentrations (10%) dropped to a minimum, they rejected the idea of the effect of the bond quality as well as the formed air voids on the electrical conductivity. They justified the reason to be the possibility of forming segregated CNT structures or the formation of a more effective CNT 3D network.

### 2.3. Thermal Anisotropy

The thermal anisotropy is reflected in the anisotropy of the thermal properties of the 3D printed polymers and polymeric composites including thermal conductivity and thermal expansion.

**Figure 6:**
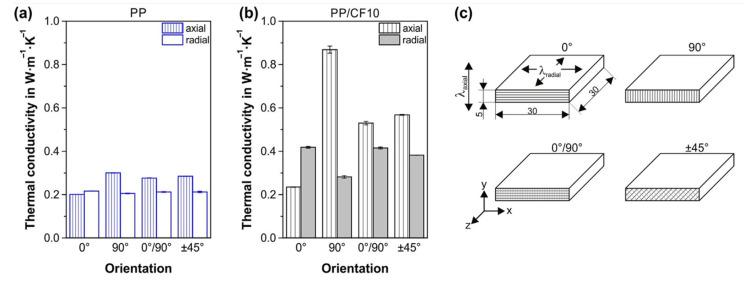
Thermal conductivity measurements based on the printing orientation for (**a**) PP and (**b**)PP/CF10. (**c**) shows the corresponding specimen dimensions and build orientations. The layers of printing are stacked in the z-direction and the heat conductivity in the axial direction (λ_axial_) is measured in the y-direction. The heat conductivity in the radial direction (λ_radial_) is also measured in the xz-plane (**c**). Adapted with permission from Ref. [74]. 2018, Elsevier.

Generally, polymers without any conductive particles cannot be considered as good thermal conductive materials. Some 3D-printed samples were tested against their thermal conductivities and for FDM-printed ABS polymer, the highest conductivity was measured to be around 0.25 W/mK [46]. Adding conductive particles with high thermal conductivities to the polymers can improve the thermal conductivity of the parts substantially. However, thermal conductivity in polymer composites might even decrease further since the anisotropy phenomena were found as those for mechanical and electrical anisotropies. FDM-printed composite parts showed different thermal conductivities when the build orientation is changed. In a study performed by Spoerk et al. [74], the difference in thermal conductivity properties of pristine and carbon fibre loaded polymers was studied when raster angle and thermal measurement orientations were changed. Although pristine polymer samples did not show great thermal conductivity values, both pristine and composite parts for a part printed with a 90° raster angle showed the best thermal conductivity when an axial measurement is performed. Although pristine polymers represented different thermal conductivities when raster angle and measurement directions were changed but the difference was not noticeable. Adding filler accentuated this discrepancy on the thermal properties of the printed parts. These results also emphasise on how adding conductive additives aligned in a specific direction to improve the thermal conductivity in that direction. Moreover, the approach of conductivity measurement can influence the conductivity results largely as the through-layer measurements can be influenced by the parameters such as air voids. Figure 6 demonstrates the thermal conductivity anisotropy in parts printed with different build orientations as discussed.

The influential parameters on the anisotropy of thermal conductivity can be related to the type, size, and orientation of the filler particles. For instance, if conductive particles are aligned with each other and do not cross this can leaves the transverse build orientation with no conductive particles. Shemelya et al. [27] reported that the thermal conductivity of the ABS/Graphite sample dropped from 0.3718 W/mK for longitudinal build orientation to 0.25171 W/mK for a transverse orientation. Using optical imaging, the authors explained that this behaviour was based on the orientation of the graphite particles as well as the carbon fibre filaments along with the print orientation. Interestingly, samples made of ABS/Carbon Fibre showed an unexpected increase in thermal conductivity from 0.20331 W/mK for samples printed in longitudinal build orientation to 0.22171 W/mK for samples printed in transverse build orientation. It is explained that the difference in thermal conductivity values is due to the alignment of the fibres along the print direction, but this justification can be true if conductivity values were higher in longitudinal build orientation, not the transverse build orientation.

In another study conducted by Baker et al. [12], the anisotropy of thermal expansion for polymers and conductive polymers were investigated at two temperatures of 20 °C and 80 °C, respectively. The coefficient of thermal expansion (CTE) of PLA, ABS, PU and Conductive PLA (C-PLA) were compared when samples were printed longitudinal or transversal. Amongst all, C-PLA showed more than 65% anisotropy in thermal conductivity while PU showed almost no anisotropy. These results first suggest that polyurethane as a flexible polymer can resist thermal expansions while other polymers such as PLA or ABS could not resist these changes. The second conclusion is that the conductive additives worked well in favour of heat transfer and caused anisotropy in thermal expansion. The reason for these changes was not addressed in this study but from the results, a range of contributing factors such as additive orientation, potentially trapped air voids and surface adhesion in forming different thermal expansion results are involved.

Prajapati et al. [29] investigated the anisotropy of thermal conductivity and the effect of some machine parameters such as air gap, raster direction and build orientation on the intensity of the anisotropy of thermal resistance for printed ABS and Polyetherimide (ULTEM) parts. With their experimental data, they found that by increasing the air gap as a machine parameter, the thermal resistance in the X raster direction, as well as transverse build orientation, increases noticeably due to the poor interfacial contact between adjacent layers. Moreover, their computed interlayer thermal resistance shows a progressive increase as the air gap size increases. This research suggests not only the increased number of air voids and their sizes act as a resistance and blocks the flow of the heat, but also the adherence of the layers drop which can lead to anisotropy in heat transfer. Although these results showed tremendous effects of generated air voids and layer adhesion on the thermal resistance of 3D printed material, the anisotropy that forms from different build orientations was not investigated thoroughly.

In another study, Elkholy et al. [75] also investigated the effects of machine parameters on the anisotropy of thermal conductivity for parts printed with the FDM method, however, the presented results were different from those findings in similar investigations. Their results showed parts printed in longitudinal build orientation had the lowest thermal conductivity when either raster width or layer height was changing. This finding is contradictory with the general findings of other work where the longitudinal build orientation shows better thermal conductivity performance.

## 3. Methods for Mitigating Material Anisotropy

The effects of material anisotropy can be minimised by altering and adjusting key factors and main control parameters of the 3D printing process, which is also depending on material type. In this section, the methods for mitigating material anisotropy are reviewed in detail.

### 3.1. Polymer and Monomer Alternation

The type of polymer is known to be an influential factor for the scale of anisotropy. Some polymers inherently do not show a great deal of anisotropy. In a recent study conducted by Allum et al. [45], it was observed that the mechanical strength in FDM-printed PLA parts was the same as the bulk material, but strain-at-fracture, toughness and specific load-bearing capacity were different for those parts printed in longitudinal and transverse build orientations.

Rodríguez et al. [47] compared the mechanical properties of two conventional polymers of ABS and PLA printed by using the FDM method. They found that PLA parts can perform much better when they were printed in transverse build orientation than ABS parts. On the other hand, the measured strength for ABS was higher when printed in longitudinal compared to PLA. These comparisons suggested that a careful selection of polymers can mitigate the anisotropy in a particular build orientation.

**Figure 7:**
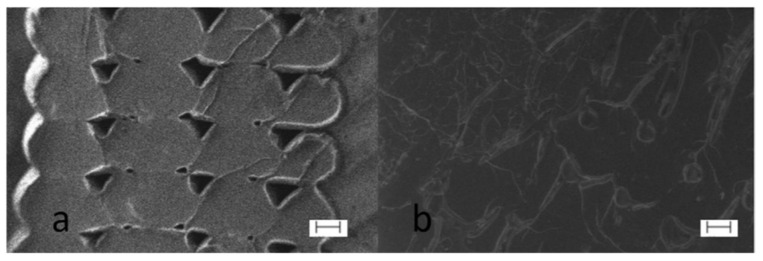
SEM images showing a decrease of air voids in printed PLA after adding low molecular weight polymer: (**a**) Neat PLA and (**b**) PLA-3%LMW polymer (scale bars equal 100 mm). Reprinted with permission from Ref. [55]. 2018, Elsevier.

In some studies, changes in polymer structure such as monomers and adding other polymers to the main polymer were proposed to increase the interlayer adhesion and consequently, improve in minimising the anisotropy effect [55,56,76]. Levenhagen and Dadmun [55] blended a high-molecular-weight polymer as the polymer base with a low molecular weight polymer as the additive. The increase of 15 to 100 per cent in maximum stress was recorded by adding 3 mol per cent of their low molecular weight polymers. The reason for this noticeable increase in material properties was related to the efficient diffusion of the low molecular polymer into inter-filament voids (see SEM images shown in Figure 7). However, this approach has the limitation of polymer selection meaning that the polymer selection is only limited to a few low and high-molecular-weight polymers.

Torrado et al. [56] added ultra-high-molecular-weight polyethylene (UHMWPE) to the blend of acrylonitrile butadiene styrene (ABS) and styrene-ethylene butadiene styrene (SEBS) to minimise the anisotropy and increase the homogeneity of the fracture surface. On the other hand, these additives caused a steep decrease in mechanical properties of the produced part compared to the pristine polymer parts. This issue could drastically influence the mechanical, electrical, and thermo-mechanical properties of the printed part in the transverse direction.

Adding a second additive polymer to the main polymer was also reported to have a negative impact on the rheology and the viscosity of the final product [55]. Torrado, Shemelya et al. investigated the feasibility of blending ABS with thermoplastic elastomer TPE [56]. Although they claimed that the lowest anisotropy and air gaps were observed in SEM images of ABS:UHMWPE:SEBS samples, but tensile test revealed that lower mechanical properties in both longitudinal and transverse printing directions were found, compared to those of the pure ABS.

Adding the crosslinking agent can result in inducing the crosslinking among printed layers and decreasing the mechanical anisotropy in parts printed in the longitudinal direction. Shaffer et al. [77] increased the toughness of FDM printed PLA parts to 1.7 folds by adding 10% of triallyl isocyanurate (TAIC) as a crosslinking agent and irradiating the part at 60 °C.

### 3.2. Adding Fillers

For the FDM printing process, in particular, one key issue is the distortion of final products, which is caused by the thermal expansion of the polymers [78]. Adding small size fillers such as micro or nano-particles can have a positive effect on the mechanical properties of these parts [3,53,79]. On the other hand, these particles in their pristine state could act as an impurity within the polymer matrices due to very weak bonding between polymer chains and these particles which potentially can cause the failure of the printed parts. Using nano-sized particles with oriented shapes and good bonding functionalities could minimise the negative effects such as material anisotropy and improve mechanical properties noticeably [3,80]. Because of the nature of 3D printing, fillers generally tend to align through the deposited lines which can increase the mechanical, thermal, or electrical conductivity properties of the parts through the lines. In a study conducted by Jia et al. [81], it is shown that based on the characteristic of FDM, rational design of the 3D printing (3DP) process can make the graphite flakes orient along the through-plane direction when the conductivity is measured to align with the deposited lines. In this work, graphite flakes were vertically aligned in maleic anhydride grafted poly (ethylene 1-octene) (POE-g-MAH) and polystyrene (PS) through FDM which resulted in through plan (transversal) thermal conductivity of 5.5 W m^−1^ k^−1^. Although lots of voids were generated during the fused deposition process, the optimal product showed a high through plan thermal conductivity because the fluent heat conductive pathways were not being blocked by the voids.

### 3.3. Altering 3-D Printing Machine Parameters

**Figure 8:**
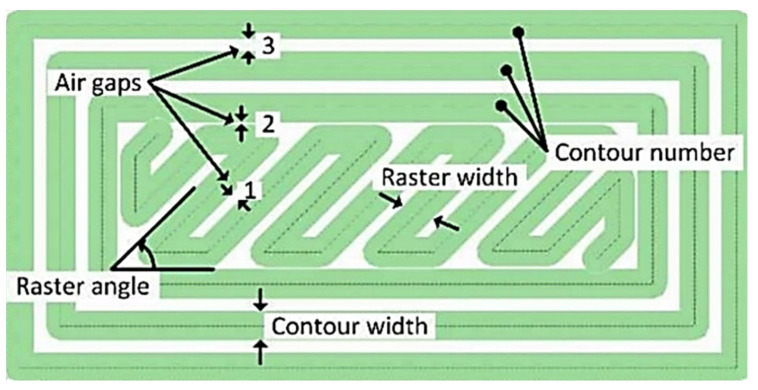
FDM processing parameters. Reprinted with permission from Ref. [82]. 2018, MDPI.

To obtain a part with acceptable performance, some machine parameters of 3D printers are considered as factors affecting the material anisotropy such as raster angle, layer thickness, build orientation, feed rate, raster width, air gap, infill density, bed and nozzle temperature, etc. [44,48]. Figure 8 schematically shows some processing parameters that can affect the properties of the parts printed by FDM.

#### 3.3.1. Raster Angle

**Figure 9:**
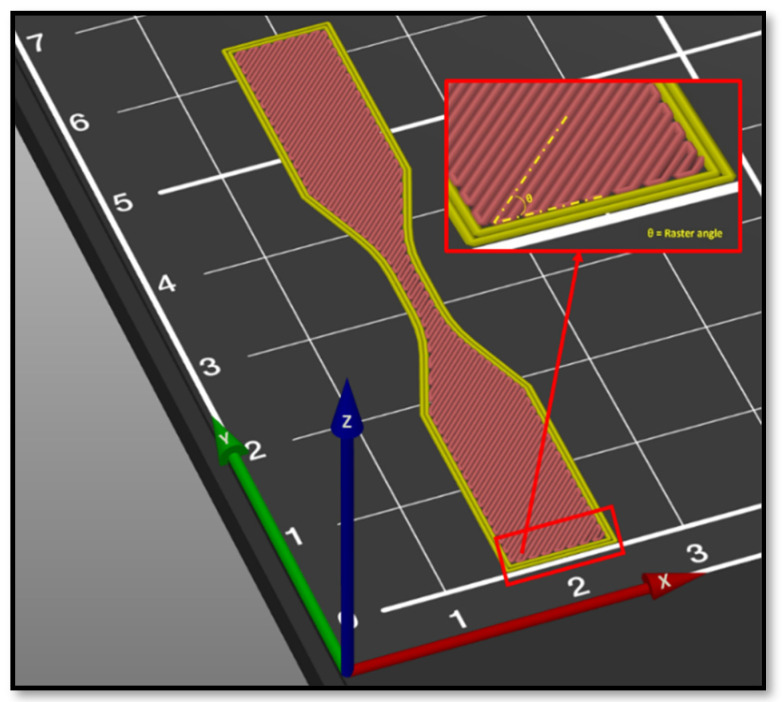
Schematic of raster angle in a 3D printed part.

Raster angle is known as the angle between the direction of the deposited beads and the *X*-axis as shown in Figure 9.

Raster angle is an influential factor in mechanical anisotropy. For poly ether-ether-ketone (PEEK) when measured along the *X*-axis the mechanical strength is 26% higher at 0° compared to 90° and around 9% higher than the strength for alternated angles between 0° and 90° [83]. For ABS, experimental results showed an increase of around 37% in 0° raster angle compared to 45° and around 43% increase compared to a 90° raster angle [48]. For PLA, however, the highest mechanical strength was recorded for 45° raster angle with around 9.37% improvement compared to 0° and 15.6% improvement compared to 90° [84]. The reason for finding 90° raster angle less favourable causing lower mechanical properties is that the mechanical load is only being carried out by the bonding between layers rather than the fibres themselves.

**Figure 10:**
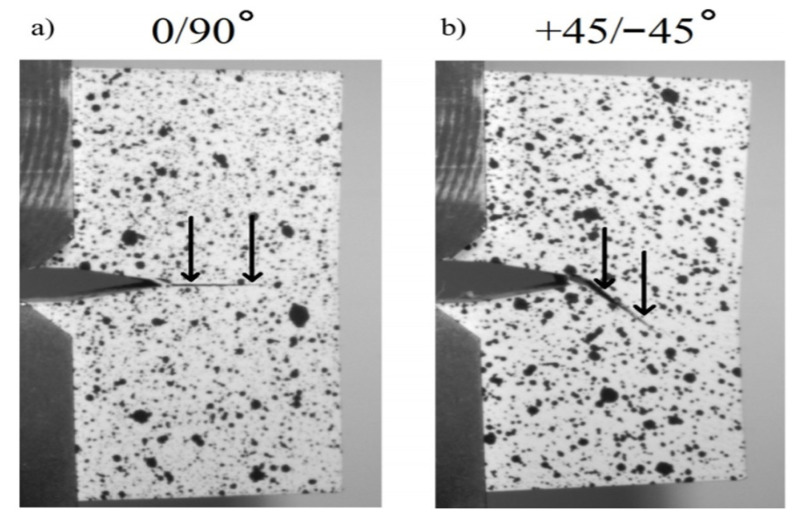
Crack propagation for the XYZ orientation containing a Y-X crack. (**a**) The in-plane crack propagation of the 0/90° samples is denoted by the black arrows. (**b**) The out-of-plane crack propagation at a 45° angle in the +45/−45° sample is denoted by the black arrows. Reprinted with permission from Ref. [85]. 2017, Elsevier.

In an interesting study conducted by McLouth et al. [85], the effect of two raster angles of ±45° and 90° in fracture toughness of ABS parts printed in three build orientations of XZY, XYZ and ZYX were investigated. When the alignment of extruded filament layers changed from parallel to perpendicular with respect to the crack plane, a 54% increase in fracture toughness was observed Results show an increase of 54% in fracture toughness when the mechanical properties were measured perpendicular to the deposited line compared to the measured value of parallel to filament direction. The results showed that the proper raster angle can effectively block the growth of the crack. Moreover, when the build orientation has layers parallel to the fracture, the raster angle can have almost no influence on the quality of the fracture toughness because the filaments are aligned with the crack growth and poor layer adhesion between two stacked rasters can cause a crack growth and consequently, failure can happen in printed part. Figure 10 shows the effect of raster angle on the angle of crack propagation.

#### 3.3.2. Printing Layer Thickness

**Figure 11:**
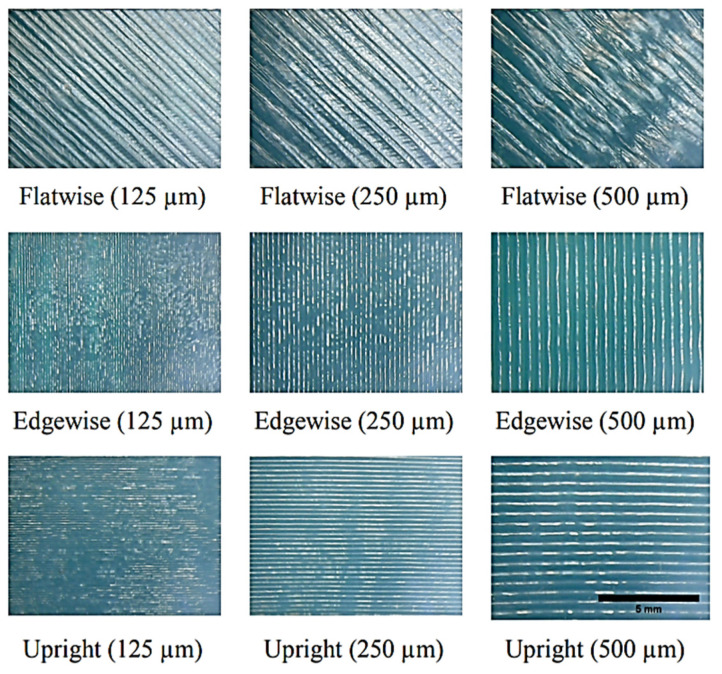
SEM images of surfaces of specimens with different print orientations and layer thicknesses (loading orientation 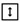) Reprinted with permission from Ref. [88]. 2017, Springer Nature.

The effect of printing layer thickness on the material anisotropy is one of the controversial topics between researchers and it demands more research [86]. Some researchers claimed that the higher the thickness, the better the mechanical properties and others draw opposite conclusions [23,44,58,87]. Kovan et al. [88] investigated the load-elongation of FDM-printed parts at three different layer thicknesses of 125 µm, 250 µm and 500 µm in transversal (upright), longitudinal (flatwise) and edgewise build orientations, respectively, as shown in Figure 11.

The results showed by increasing the layer thickness, the difference in mechanical properties for different build orientations becomes more obvious. The sample with 500 µm layer thickness showed the highest elastic modulus and bonding strength when printed at flatwise (longitudinal) build orientation. Furthermore, it is found the edgewise orientation had the highest adhesion strength in lower layer thicknesses.

Sood et al. [89] reported that the tensile strength first dropped and then increased by increasing the layer thickness. This behaviour was explained based on the distortion produced by heat gradient towards the bottom layers which cause a decrease in layer adhesion and lower mechanical strength. As the layer thickness increases, a smaller number of layers will be required and the distortion effect is minimised and hence, the material strength increases.

**Figure 12:**
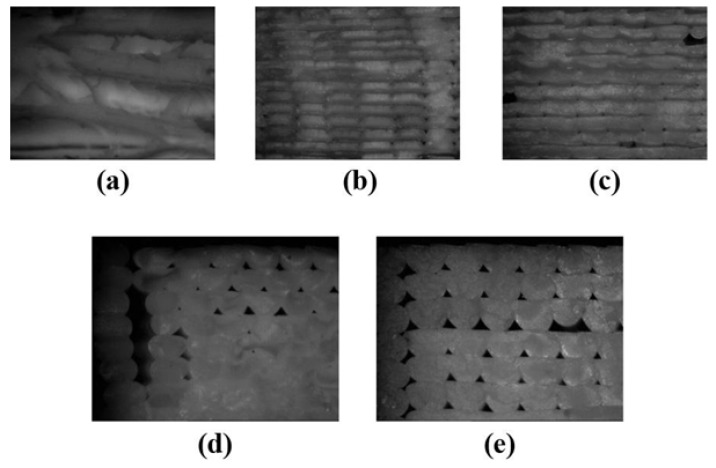
Microscopic images of the fractured surface with different printing layer thicknesses: (**a**) 100 µm, (**b**) 150 µm, (**c**) 200 µm, (**d**) 250 µm, and (**e**) 300 µm. Reprinted with permission from Ref. [92]. 2018, Elsevier.

In contradiction, Torres et al. believe that by increasing the layer thickness the mechanical strength will decrease as hypothetically, either thinner layers makes smaller voids or more layers can bear a greater deal of force and resist deformation [90]. Lee et al. also considered the lowest thickness as the optimal thickness for the maximum mechanical strength of FDM produced samples (0.178 mm) [91]. Figure 12 shows the effect of layer thickness on the formation of air voids. It should be noted that smaller layer thicknesses might reduce the sizes of air voids which results in decreasing the anisotropy, but it will increase the printing time.

#### 3.3.3. Build Orientation

**Figure 13:**
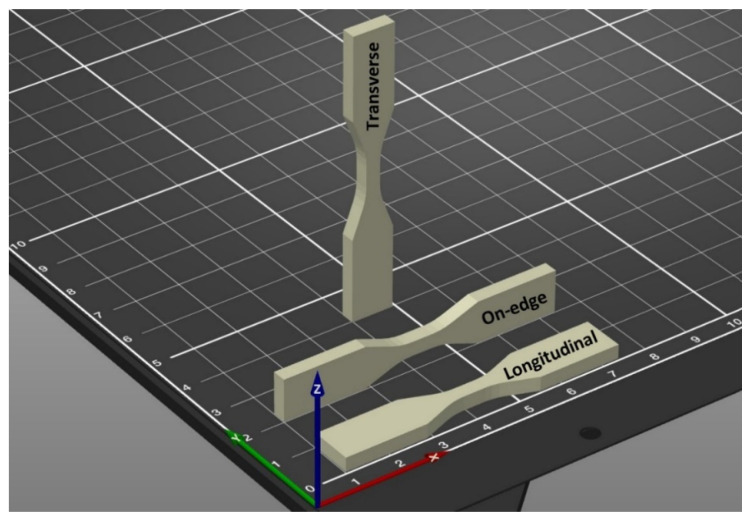
Build orientation modes.

To optimally design the FDM printed parts with the desired strength, many researchers claimed that the build orientation has the most important role in the mechanical properties of the final parts [16,44,79]. There are three different build orientations of flat (longitudinal), on-edge (sidewise) and upright (Transverse). For the “Flat” mode (XYZ direction) the sample is faced down on the printing bed and is being printed parallel to the loading direction. The second is called the “on-edge” mode (XZY direction) which the sample prints on one edge parallel to the load direction and the third is the “upright” mode (ZYX direction) which layers are deposited perpendicular to the load direction [44,88]. Figure 13 shows different print orientations of XYZ, XZY and ZYX.

The majority of the researchers reported that the best printing orientation is the one along the direction at which the mechanical strength is applied and the worst properties measured were related to the samples printed in transverse build orientation [1,86,88,92,93]. However, the goal for many researchers up to this moment was to minimise this difference to an acceptable level so the final parts could present unified multidirectional properties like the ones produced with the conventional methods such as mould injection or thermal press. As is stated in the previous sections of this review, build orientation not only affects the mechanical properties of the printed parts but also has a vital effect on the final thermal and electrical conductivity properties of the printed parts. For instance, Chacón et al. [44] reported that the ultimate tensile strength for the parts printed at the upright direction was 78% to 37% lower than those of the samples printed on edge or flat direction. Similar observations obtained by Zhang et al. [38] and Shemelya et al. [27] on lower electrical and thermal properties in parts printed in transverse orientation were reported, respectively. These orientation preferences limit the performance and workability of the printed part. Therefore, a method that could eliminate the effect of orientation on the properties of the parts produced by FDM is highly desirable.

#### 3.3.4. Feed Rate

Feed rate is known as the volumetric measure of the polymer extruded from the tip of the extruder nozzle. Increasing and decreasing the feed rate will affect the speed and time of part production. The effects of feed rate on mechanical properties were studied by some researchers while its effect on anisotropy was not studied extensively up to this moment [44,65,94,95]. According to a published work by Chacón et al. [44], in a fixed layer thickness of 0.06 mm and the feed rate of 20 mm/s, the mechanical anisotropy was measured to be around 60 per cent while when the feed rate was increased to 80 mm/s, the mechanical anisotropy increased to almost 77.7 per cent. Interestingly, at a feed rate of 20 mm/s, by increasing the layer thickness from 0.06 mm to 0.24 mm, the mechanical anisotropy was measured to be 35.7 per cent. The same trend and measuring mechanical anisotropy at 38.4 per cent were observed when the feed rate was 80 mm/s and the layer thickness was 0.24 mm. This indicates not only the layer thickness can have a tremendous influence on the mechanical anisotropy, but also by increasing the feed rate, more mechanical anisotropy can be measured.

### 3.4. Post-Processing Heat Treatment

Since one of the most important parameters influencing the anisotropy in parts printed via AM methods is the low bonding between the adjacent printed layers, improving this feature is a matter of attention in recent publications. In the following section, different proposed processing methods to rectify the anisotropy are reviewed.

In parts produced by the FDM method, there is a possibility of the formation of weak bonds between adjacent deposited lines and consequently delamination because of the shrinkage and residual stress. Therefore, it is very important to monitor the variation of temperature while printing, after the filaments are deposited and cooling down [48,96,97].

Heat treatment can have a positive influence on minimising internal stress and anisotropy if the temperature is selected properly. Two methods of post-processing heat treatment and in-processing heat treatment are mentioned in the literature. For post-processing heat treatment, annealing was suggested by few researchers. Sathish Kumar et al. [98] suggested that annealing can be an effective method to improve mechanical properties of samples made of Polyethylene Terephthalate Glycol (PETG) and Carbon Fibre reinforced Polyethylene Terephthalate Glycol (CFPETG) by 8% and 22%, respectively. Based on their research, they found that this improvement can be attributed to residual stresses, which are created during the rapid cooling and heating of the printing process. By subjecting the samples to heat, it is suggested that residual stresses may become free.

The temperature of annealing has a great influence on the final part. For instance, Knop et al. defined annealing as subjecting the specimens to further heat treatment after printing for a particular period. In their study, Poly amid 12 (Nylon) parts was heat-treated for 180 min at 135° and the results showed improvement in tensile strength as well as flexural strength because of degradation of internal stress and post-crystallisation [36]. In their case, the selected temperature is almost two times higher than the Tg of Nylon and based on their DSC results, after the heat treatment, bigger and more oriented crystallites were formed, which can generate better forces between polymer chains.

In another study, Torres et al. stated that the PLA samples have shown better tensile strength results compared to untreated PLA when heated at temperatures between 65–80 °C (which is just slightly above the Tg of PLA) [90]. However, the difference becomes more obvious when samples were heated to 100 °C. In another study, Kantaros and Karalekas studied the effects of thermal treatment on compressive residual strains of their ABS parts and they found that the samples treated with temperatures lower than the Tg of ABS showed no changes at all [99]. Although post-processing can have a positive influence on the properties of the FDM printed parts, but, the fact that it applies higher costs and labour time on the shoulders of the producer is undeniable. Therefore, other ideas to promote in-process improvements are more favourable.

Few published works attempted using the in-process methods to tackle anisotropy in the Z direction and the possible ways to reduce its negative effects on the material properties of the finished part. For instance, Ravi et al. and Kishore et al. proposed the use of heat in form of light to heat the surface of the previously printed layer right before the new layer is going to be deposited and Partain used forced air to heat the whole sample [39,100,101]. Ravi et al. used localised near IR laser beams to supply the heat needed to soften up the previous layer. They reported a 50% improvement in the interlayer bond strength after applying heat. The same method was adopted by Striemann et al. to manufacture 3d printed short carbon-fibre-reinforced polyamide samples in transverse build orientation. Their infrared preheating system (IPS) was claimed to be effective in maintaining the temperature of the interlayer contact zone above the glass transition which led to minimised pore sizes and improved interlayer adhesion [102]. Although Kishore et al. and Partain reported improvements in material properties of their printed parts since their method of heat emission is not focused, the produced parts are more susceptible to part dimensional and structural inaccuracy and deformation [100]. In the case of the localised energy beam laser, the drawbacks are the high costs of the laser parts as well as the high temperatures for localised and partial melting.

#### Air Gap

The air gap is also another adjustable machine factor that is influential on mechanical anisotropy. An air gap is a distance between two adjacent rasters, between a raster with and a contour or between two adjacent contours [82]. The zero air gap defines as no space between each raster; negative air gap means there is an overlap between the rasters and positive means there is a space between rasters and they do not touch [33]. The negative air gap can improve the integrity of the part and also facilitate diffusions between adjacent layers [17] but it can also result in having a part with uneven surfaces, dimensional inequality and higher printing times [103,104]. A positive air gap, on the other hand, can help improve the printing time but it also can cause having a loosely packed part with an uneven surface and low mechanical properties [104]. Although the air gap can be set as zero via the settings of the FDM machine to theoretically have no air gaps, the polymer fibre geometry inherently can cause forming triangular or rhombus air voids in each layer and/or between layers [48,63].

**Figure 14:**
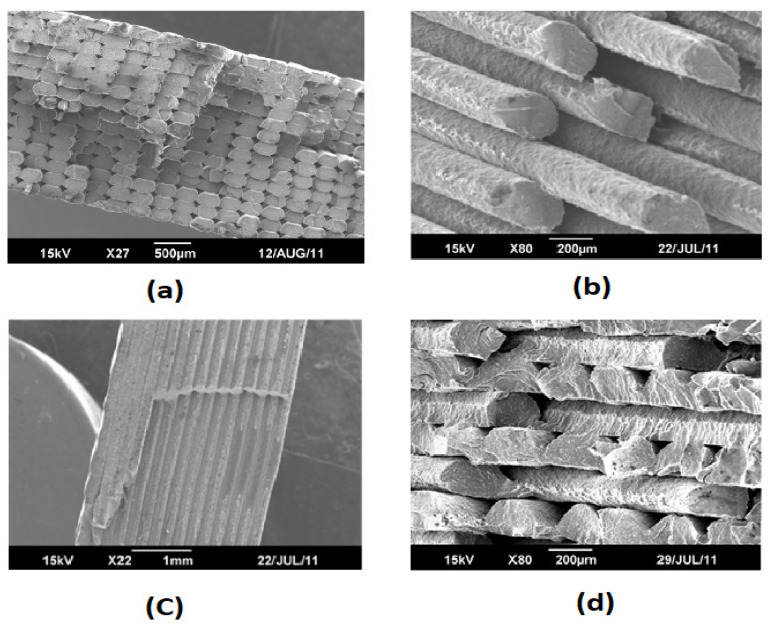
SEM image from fractured surface (**a**) 0°, (**b**) 45°, (**c**) 90° and (**d**) +45°/−45°. Reprinted with permission from Ref. [48]. 2011, IntechOpen.

These voids decrease the physical cross-sectional area of the parts and they not only influence the effective elastic moduli and effective tensile strength of the part but also can greatly decrease the thermal and electrical conductivity especially in the *Z*-axis (printing direction) compared to X-direction [28,29]. This void formation and decrease in mechanical properties can result in delamination of the layers after the part is being printed [1]. Figure 14 shows the air void formation in FDM parts independent of raster angles.

### 3.5. Other Related Factors and Parameters

Some other factors and parameters such as raster width [92,105], nozzle and bed temperatures [40], infill density [106,107] were reported in the literature. Although these factors were addressed to be influential factors on the mechanical properties of the printed parts, their influences on the anisotropy of the parts was not specifically investigated yet.

**Figure 15:**
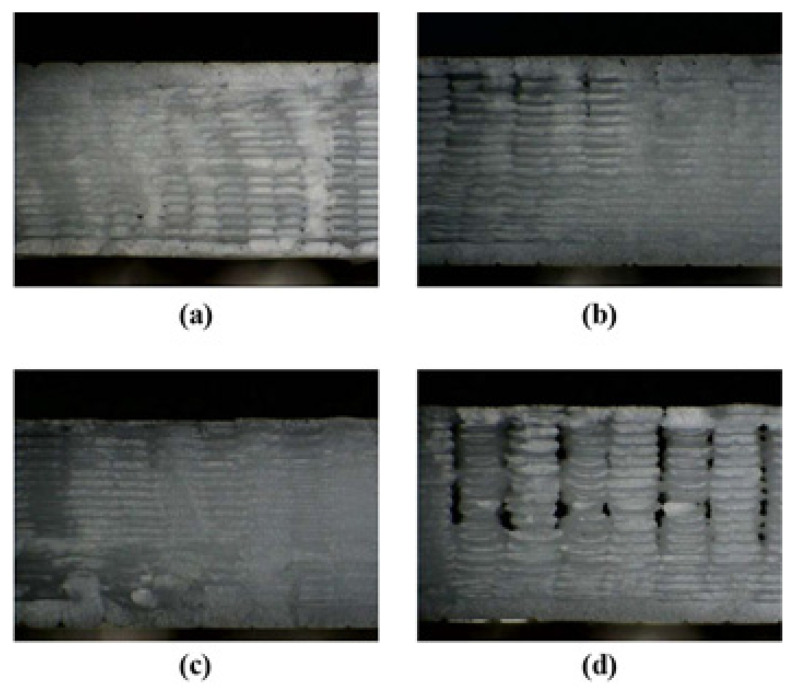
Microscopic images of fractured surface at raster widths of (**a**) 400 µm, (**b**) 500 µm, (**c**) 600 µm and (**d**) 700 µm Reprinted with permission from Ref. [92]. 2018, Elsevier.

As for the raster width, Rajpurohit and Dave [92] and Coogan and Kazmer [105] observed an increase in tensile strength by increasing the raster width up to a certain extend. The reason was claimed to be related to the higher thermal mass of raster, lower rates of heat dissipation and as a result, more time for layer diffusion and better layer adhesion. After a certain increment in layer width, the air void formation between raster layers causes a huge drop in mechanical properties which consequently leads to crack initiation, propagation and failure of the part [92]. Figure 15 shows the effect of raster width increase on the formation of the air voids.

Nozzle and bed temperatures are known to be two great influencing factors on the neck growth in polymeric parts. Materials with higher melting points require higher nozzle and bed temperatures to minimize warpage and better adherence to the bed surface. For instance, Aliheidari et al. investigated the effect of both nozzle and bed temperatures and found that interlayer fracture resistance has increased by 15 per cent when nozzle temperature increased from 220 °C to 240 °C and 17 per cent when print bed temperature increased from 85 °C to 105 °C [40].

Infill density is known as the amount of filament material that is printed inside the designed part. By changing the infill density, the properties of the final printed part can be affected. Moreover, different infill types and patterns can generate different effects on the mechanical, thermal, and electrical properties of the parts. Singh et al. found that the effects of infill density on the thermal and electrical conductivity of ABS-Graphene composite can be increased by 50% and 34% when the infill was increased from 50% to 100%, respectively [106]. In another study, Aw et al. recorded a 17% increase in measured tensile strength for their ABS/ZnO samples by increasing infill from 50 to 100 per cent and also indicated that the increase of strength is a result of the increase in infill density and also the bond between two adjacent layers which could lead to minimising the air gaps.

The other challenge is the postprocessing of the additively manufactured materials via heat treatment. The heat treatment can reduce the porosity but also may generate unwanted dimension changes. It is possible for researchers to develop a hybrid additive manufacturing-thermal press technology with optimal process control to obtain high-quality additively manufactured materials.

To relate all these parameters to the anisotropy of printed parts, investigations in parts printed in directions perpendicular to the applied force is required. Additionally, although these researchers did not investigate the anisotropy independently, their results show discrepancies in layer adhesion and air voids which could potentially affect the properties of the printed parts when measurements are perpendicular to the layer formation.

## 4. Research Challenges

This review summarises the efforts in projecting the phenomenon of anisotropy in mechanical, thermal, and electrical properties of parts printed with AM methods. The most important challenge is recognised as to minimise or eliminate the gap in mechanical strength, thermal and electrical conductivity values when changing the build orientation and obtain similar properties to the part made by traditional manufacturing processes including mould injection and thermal press.

To tackle mechanical anisotropy, a wide range of ideas from altering polymers to adding polymers and monomers and applying heat while printing or post-processing was suggested. However, limiting the printing process to only a few polymers or adding a specific monomer to minimise the anisotropy level may not be the ultimate solution. Moreover, adding further steps to the printing process such as post-processing might potentially increase the time and cost of the final product.

For thermal and electrical conductivities, adding thermally or electrically conductive micro or nano-additives were also suggested to be influential in increasing the conductivity of the polymer part. However, because of the way that the polymers are deposited especially in the FDM printing method, there were cases found that the additives are stacked and oriented through the deposition direction and it caused an increase in thermal or electrical anisotropies when the properties are measured interlayer or through the layer. Additionally, adding additives in high concentrations can help to increase the thermal or electrical conductivities but from a certain level, it can destroy the integrity of the polymer chains and consequently, diminishing the mechanical properties of the parts.

Amongst contributing factors on increasing the thermal or electrical anisotropies in parts, the number and size of the air voids, as well as the layer adhesion in parts printed via FDM, were known to be some of the important reasons for anisotropy formation. The reason is that the formed air voids can act as an insulator and stops transferring heat or electricity through layers.

Some machine-related parameters such as raster angle, layer thickness, build orientation and feed rate were also considered as the influential parameters but finding an optimum condition that could serve a vast range of polymers remains a mystery.

## 5. Recommendations on Future Work

In addition to the above-mentioned challenges, there is also a lack of a comprehensive study which investigates the anisotropy of combined mechanical, thermal and electrical properties (thermo-electro-mechanical anisotropy) of 3D printed polymer composites. To achieve this, a good understanding of affecting factors on layer adhesion and formation of air voids is required. This proposed study could lead to a method that eliminates or mitigates the unwanted thermo-electro-mechanical anisotropy.

Furthermore, the following future work can be recommended to further mitigate the material anisotropy of 3D printed polymer or polymer composites via: (a) development of the hybrid additive manufacturing with combining the conventional manufacturing technology, for example, FDM–Thermal Press technology to reduce their porosity and increase bonding between layers of the 3D printed materials; (b) Optimal design of 3D printing process for polymers and polymer composites with appropriate settings on its control parameters for printing high-quality 3D printed materials; (c) development of effective post-processing, e.g., heat treatment, which can greatly mitigate the material anisotropy in an optimal manner; and (d) 3D printing of polymer composites with appropriate additives and nano-additives, which could increase bonding between layers of the 3D printed materials.

## 6. Concluding Remarks

A review on material anisotropy of the 3D printed polymers and composite parts including mechanical, electrical, and thermal anisotropies and the main factors known to be influential on anisotropy has been presented in this paper. It was discussed that not only the mechanical properties but also the electrical and thermal conductivity of the printed parts are influenced by the anisotropy phenomenon.

To minimise the negative influence of anisotropy in 3D printed parts, different approaches and techniques such as polymer and monomer alteration, additive incorporation, chemical treatment, optimally changing 3D printer’s process and control parameters and post-processing heat treatments were developed by researchers with merits and demerits. Although some methods could minimise the level of anisotropy to a certain amount, a method that could effectively eradicate it is still required. Moreover, in regard to anisotropy in surface electrical and thermal conductivity more focus is needed to understand the effect of parameters such as build orientation, layer thickness, layer width, air gap and temperature on thorough layer conductivity and final conductivity of the built material.

## Figures and Tables

**Table 1:** Anisotropy control factors and parameters.

Material Factors	3D Printing Process Control Parameters	Process Factors of 3D Printing or Additive Manufacturing
Polymer and monomer alternation [31,32] *	Raster angle [33,34] *	Heat treatment [35,36] **
Adding additives [27,37,38] ***	Layer Thickness [33] **	Using heat gun, light, and laser [30,39] *
Chemical treatment [40,41,42]	Build orientation [14,43] ***	
	Feed rate [44] *	
	Raster width [38,45]	
	Bed and nozzle temperatures [35,45] ***	
	Infill density [46,47] ***	
	Air gap [38] *	

Zero stars are the factor with the least influence on anisotropy, * low influence, ** medium influence and *** high influence.

**Table 2:** Summary of anisotropy in polymeric parts printed by different AM methods.

Material	Printing Method	Anisotropy Type	Anisotropy before Modification	Anisotropy after Modification	Proposed Mitigation Method	References
Acrylonitrile butadiene styrene (ABS)	FDM	Mechanical	26%	-	-	[50]
Nylon	LS	Mechanical	16%	-	-	[18]
ABS	FDM	Mechanical	25%	-	-	[51]
Polyaniline (PA12)	SLS	Mechanical	3%	-	-	[51]
ABS M30 ABS M30i	FDM	Electrical	75% 26%	-	-	[52]
DuraForm-HST Nylon EX	SLS	Electrical	29% 17.5%	-	-	[52]
VeroBlue VeroAmber	Polyjet	Electrical	53% 71%	-	-	[52]
Epoxy/Fumed silica (FS) Epoxy/Nanoclay (NC) Epoxy/Silicon carbide (SiC)+FS Epoxy/SiC+NC	DIW	Mechanical	1.2% 31.6% 39.6 48.1	-	-	[53]
ABS ABS+ short carbon fibres (SCF)	FFF	Mechanical	37.7% 82.3%	-	-	[54]
Polylactic acid (PLA)	FDM	Mechanical	77%	35.7%	Selecting on edge or flat build orientation, changing feed rate and layer thickness	[44]
ABS PLA	FDM	Mechanical	88% 28%	-	Careful selection of build orientation to load direction	[47]
PLA	FDM	Mechanical	166%	33%	Addition of low molecular weight polymer to high molecular weight polymer	[55]
ABS	FDM	Mechanical	47.7%	21.97% at 75:25:10 ratio of ABS:UHMWPE:SEBS	Addition of a variety of additives such as Titanium dioxide, Zinc oxide, Strontium titanate, Aluminium oxide, Styrene-Ethylene-Butadiene-Styrene and Ultra-high-molecular-weight polyethylene to decrease anisotropy	[56]
ABS High impact polystyrene (HIPS)	FDM	Mechanical	34.63%	0.5%	Changing the type of polymer from ABS to HIPS	[16]
VeroWhitePlus RGD 835	PolyJet	Mechanical	7.2% at Flat X and Vertical X	-	-	[57]
S-PRO X-GREEN ABS	SLA	Mechanical	17.8% 56.7% −3.1%	-	-	[58]
Poly (butylene terephthalate) (PBT)/Carbon nanotube (CNT)	FDM	Electrical	32%	-	-	[28]
ABS/Graphite ABS/Carbon fibre (CF)	FDM	Thermal	32% 9%	-	-	[27]
Conductive PLA Conductive ABS Conductive PU	FDM	Thermal	65% 40% 0%	-	Proper selection of polymer can influence thermal conductivity	[12]

## Data Availability

The data presented in this study are available upon request from the corresponding author.
